# Extensive chloroplast genome rearrangement amongst three closely related *Halamphora* spp. (Bacillariophyceae), and evidence for rapid evolution as compared to land plants

**DOI:** 10.1371/journal.pone.0217824

**Published:** 2019-07-03

**Authors:** Sarah E. Hamsher, Kyle G. Keepers, Cloe S. Pogoda, Joshua G. Stepanek, Nolan C. Kane, J. Patrick Kociolek

**Affiliations:** 1 Department of Biology, Grand Valley State University, Allendale, Michigan, United States of America; 2 Annis Water Resources Institute, Grand Valley State University, Muskegon, Michigan, United States of America; 3 Department of Ecology and Evolutionary Biology, University of Colorado, Boulder, Colorado, United States of America; 4 Department of Biology, Colorado Mountain College, Edwards, Colorado, United States of America; 5 Museum of Natural History, University of Colorado, Boulder, Colorado, United States of America; Austrian Federal Research Centre for Forests BFW, AUSTRIA

## Abstract

Diatoms are the most diverse lineage of algae, but the diversity of their chloroplast genomes, particularly within a genus, has not been well documented. Herein, we present three chloroplast genomes from the genus *Halamphora* (*H*. *americana*, *H*. *calidilacuna*, and *H*. *coffeaeformis*), the first pennate diatom genus to be represented by more than one species. *Halamphora* chloroplast genomes ranged in size from ~120 to 150 kb, representing a 24% size difference within the genus. Differences in genome size were due to changes in the length of the inverted repeat region, length of intergenic regions, and the variable presence of ORFs that appear to encode as-yet-undescribed proteins. All three species shared a set of 161 core features but differed in the presence of two genes, *serC* and *tyrC* of foreign and unknown origin, respectively. A comparison of these data to three previously published chloroplast genomes in the non-pennate genus *Cyclotella* (Thalassiosirales) revealed that *Halamphora* has undergone extensive chloroplast genome rearrangement compared to other genera, as well as containing variation within the genus. Finally, a comparison of *Halamphora* chloroplast genomes to those of land plants indicates diatom chloroplast genomes within this genus may be evolving at least ~4–7 times faster than those of land plants. Studies such as these provide deeper insights into diatom chloroplast evolution and important genetic resources for future analyses.

## Introduction

Diatoms are single-celled eukaryotic algae with silica cell walls. They play an important role in global O_2_, CO_2_, and silica cycling [1,2] and have the most efficient Rubisco known [3]. Diatoms are the most diverse group of eukaryotic algae [4] with an estimated 100,000 species [5] and occupy many niches in marine and freshwater environments [6].

*Halamphora* (Cleve) Levkov [7] is a recently described genus composed of species formerly assigned to the genus *Amphora* Ehrenberg ex Kützing. Members of the genus occur across a wide ecological spectrum including fresh to hypersaline habitats [8] from the tropics to the polar regions [9,10], and are known to be prodigious oil producers [11,12]. Although diverse, recent taxonomic treatments [7,8] and broadly sampled molecular phylogenetic analyses [13,14] have combined to make *Halamphora* one of the most well studied groups of diatoms in terms of phylogenetic systematics.

Despite diatom diversity and importance as primary producers in most aquatic ecosystems, the chloroplast genomes of only 40 species have been analyzed to date and taxon sampling has primarily focused on the ‘polar’ centric diatoms (45% of published chloroplast genomes; [15,16,17]). Outside of this group, no two species from within the same genus have been examined prior to this study. As the cost of genomic sequencing has decreased [18], it has become more feasible to examine in greater detail the genetic makeup of biologically important, non-model organisms. This allows for salient comparisons and insight into evolutionary lifestyle mechanisms at the genetic level. Finer scale taxonomic sampling in non-model organisms, such as within genus-level comparisons, elucidates the time-scales of evolutionary processes that may occur at rates too rapid to yield meaningful comparisons at more sparse taxonomic sampling regimens.

Therefore, the purpose of this study is to explore the phylogenetic and genomic relationships between three closely related *Halamphora* species (*H*. *americana* Kociolek in Kociolek et al., *H*. *calidilacuna* Stepanek & Kociolek, and *H*. *coffeaeformis* (C.Agardh; Levkov)). We then compared overall genomic content between these newly sequenced and annotated genomes to currently published diatom plastid genomes. Strikingly, *Halamphora* demonstrates high levels of gene rearrangement in comparison to the genus *Cyclotella*. The levels of gene rearrangements in the genus *Halamphora* are comparable to the level observed in much older-diverging lineages of the major groups of land plants (dicots and monocots).

## Materials and methods

### Ethics statement

No permits were required for collection of benthic samples in Salt Alkaline Lake, ND or Blue Lake Warm Spring, UT. No specific permissions were required for these locations/activities. Both sites are publicly accessible and there are no regulations regarding the collection of algae from these sites. Field studies did not involve endangered or protected species.

### Isolate collection and culturing

Environmental samples containing *H*. *americana* and *H*. *coffeaeformis* were collected from Salt Alkaline Lake, ND in 2011 and samples containing *H*. *calidilacuna* were collected from Blue Lake Warm Spring, UT in 2012 (Table 1). Conductivity and pH measurements were recorded from the sites at the time of collection using a YSI 556 multi-probe (YSI Incorporated, Yellow Springs, Ohio, USA). Individual cells were isolated into monoculture by micropipette serial dilution and grown in artificial brackish water medium created using the sea salts Instant Ocean (Spectrum Brands, Inc., Blacksburg, Virginia, USA). Conductivity of the medium was adjusted to 10 mS cm ^-1^ to approximate the conductivity at the collection sites (Table 1) and added macro- and micronutrients were based on those of WC media [19] with the Na_2_SiO_3_ concentration increased to 56.85 mg l^-1^. Cultures were maintained at ~25°C, under fluorescent illumination with a 12:12 light cycle at an irradiance of ~50 μmol cm^-2^ S^-1^.

**Table 1:** Isolates utilized in this study.

Taxon	Voucher	Collection Locality	Latitude (°N)	Longitude (°W)	pH	Conductivity (mS cm^-1^)
*H*. *americana*	JPK7977-AMPH100	Salt Alkaline Lake, Kidder Co., ND, USA	46.95092	99.53915	8.89	9.811
*H*. *calidilacuna*	JPK8506-AMPH118	Blue Lake Warm Spring, Tooele Co., UT, USA	40.50257	114.0336	7.60	9.319
*H*. *coffeaeformis*	JPK7977-AMPH101	Salt Alkaline Lake, Kidder Co., ND, USA	46.95092	99.53915	8.89	9.811

### DNA extraction, library preparation, and sequencing

Cultures were harvested by centrifugation and DNA was extracted using the Qiagen DNeasy Plant Mini Kit (Qiagen, Crawley, UK) following manufacturer’s protocols.

Library preparation and sequencing followed Pogoda et al. [20]. Briefly, genomic libraries were prepared using Nextera XT DNA library prep kits (Illumina). The protocol calls for 1 ng total of input DNA and each gDNA sample was diluted to the appropriate concentration using a Qubit 3.0 fluorometer (ThermoFisher Scientific). Each sample was barcoded by the unique dual index adapters Nextera i5 and i7. Resulting libraries were cleaned using solid phase reversible immobilization (SPRI) to remove fragment sizes less than 300 base pairs via an epMotion 5075TMX automated liquid handling system. Sample quality control (QC) was conducted prior to normalizing the loading concentration of pooled samples to 1.8–2.1 pM with 1% PhiX control v3 added (Illumina). Samples were processed for paired end 151 base pair reads on the Illumina NextSeq sequencer at the University of Colorado’s BioFrontiers Institute Next-Generation Sequencing Facility in Boulder, Colorado. All wet lab work was performed in the Department of Ecology and Evolutionary Biology at the University of Colorado, Boulder.

### Assembly and annotation

Raw de-multiplexed data were sub-sampled to approximately 2 GB per sample and trimmed using Trimmomatic-0.36 with the following parameters: ILLUMINACLIP:NexteraPE-PE.fa:2:20:10MINLEN:140 LEADING:20 TRAILING:20 [21]. Resulting fastq files were *de novo* assembled using SPAdes v3.9 with the following parameters: SPAdes-3.9.0-Linux/bin/spades.py—careful -k 35,55,85 [22]. Diatom chloroplast contigs were identified using command-line BLAST against *Phaeodactylum tricornutum* (accession EF067920) and confirmed using a web BLAST. Annotations were initiated in DOGMA [23] and completed in NCBI’s Sequin 15.10 (Bethesda, MD) using the protein-coding sequence of 23 diatom chloroplast genomes as references (S1 Table). Putative features unique to these genomes (or not found in the reference genomes) were identified using NCBI’s ORF Finder (Bethesda, MD). Annotated plastid genomes are available in GenBank using accession numbers MK045450 –MK045452.

Genome content was visualized using OGDraw v1.2 [24]. Total sequence length of protein coding genes (CDS), transfer RNAs (tRNAs), ribosomal RNAs (rRNAs), and non-coding DNA was calculated as a sum of the total content of each type of feature. Web BLAST was used to determine if unique ORFs identified using NCBI’s ORF Finder contained any sequence similarity to ORFs found in other species’ chloroplast genomes. A gene was considered present if there was an appropriate, full length BLAST hit to a reference diatom chloroplast. Otherwise, it was assumed absent. In addition, gene density (number of protein coding genes / genome size) was calculated [25].

### Chloroplast phylogeny

Twenty molecular markers (*psaA*, *psbC*, *petD*, *petG*, *atpA*, *atpG*, *rbcL*, *rbcS*, *rpoA*, *rpoB*, *rps14*, *rpl33*, *rnl*, *rns*, *ycf89*, *sufB*, *sufC*, *dnaK*, *dnaB*, *clpC*) from 26 species were aligned (S1 File) using the MAFFT algorithm [26], manually edited as necessary, and partitioned by gene and codon position (except for ribosomal DNA regions). Maximum likelihood analysis with 50 independent tree searches and 1000 rapid bootstrap replicates was performed in RAxML [27] with the graphical user interface raxmlGUI ver. 1.2 [28] using the GTR+Γ+I model of evolution.

Pairwise genetic distances utilizing a Jukes-Cantor model of evolution were calculated for the twenty-marker concatenated alignment using R-Studio v1.1.456 using the ‘dist.dna’ function in the R package ape [29].

### Synteny

Two separate alignments of biraphid pennate and thalassiosiroid diatoms and resulting locally collinear blocks (LCBs) were estimated with MAUVE 2.4.0 [30] after eliminating one copy of the inverted repeat (IR). Rearrangement distances between LCBs were measured using GRIMM 2.02 [31].

## Results and discussion

### General features

*Halamphora* chloroplast genomes varied in size from ~120–150 kb (Table 2), representing a size difference of ~20% within the genus. Despite this difference in size, they contained similar GC content (~30%; Table 2). The *Halamphora* genomes contain two canonical inverted repeats (IRs) separated by a small (SSC) and a large single-copy (LSC) region, a structure these genomes share with other diatoms (e.g., [16,32]), some red algae (e.g., [33]), glaucophytes (e.g. [34]), some green algae (e.g., [35]), and most land plants (e.g., [36]).

**Table 2:** Quantification of features in three *Halamphora* species, including % non-coding sequence, % GC, the number of ORFs (the number of ORFs shared by > 1 *Halamphora* genome), and gene density.

Taxon	LSC	SSC	IR length	Genome size	tRNA	rRNA	CDS^a^	Feature encoding	Non-feature encoding	% non-coding	GC (%)	ORFs (shared)	Gene Density^b^
*Halamphora americana*	77,289	44,724	10,269	142,551	2202	8720	95,428	106,350	36,201	25.4	32	7 (1)	0.92
*H*. *calidilacuna*	82,227	49,698	9407	150,739	2199	8710	102,024	112,933	37,806	25.1	32	15 (1)	0.88
*H*. *coffeaeformis*	64,938	41,485	7752	121,927	2199	8708	91,032	101,939	19,988	16.4	31	2	1.07

LSC, large single copy region; SSC, small single copy region; IR, inverted repeat; CDS, coding sequence; ORF, open reading frame.

^a^Includes coding sequence and intronic ORFs

^b^Number of protein-coding genes / genome size

**Fig 1:**
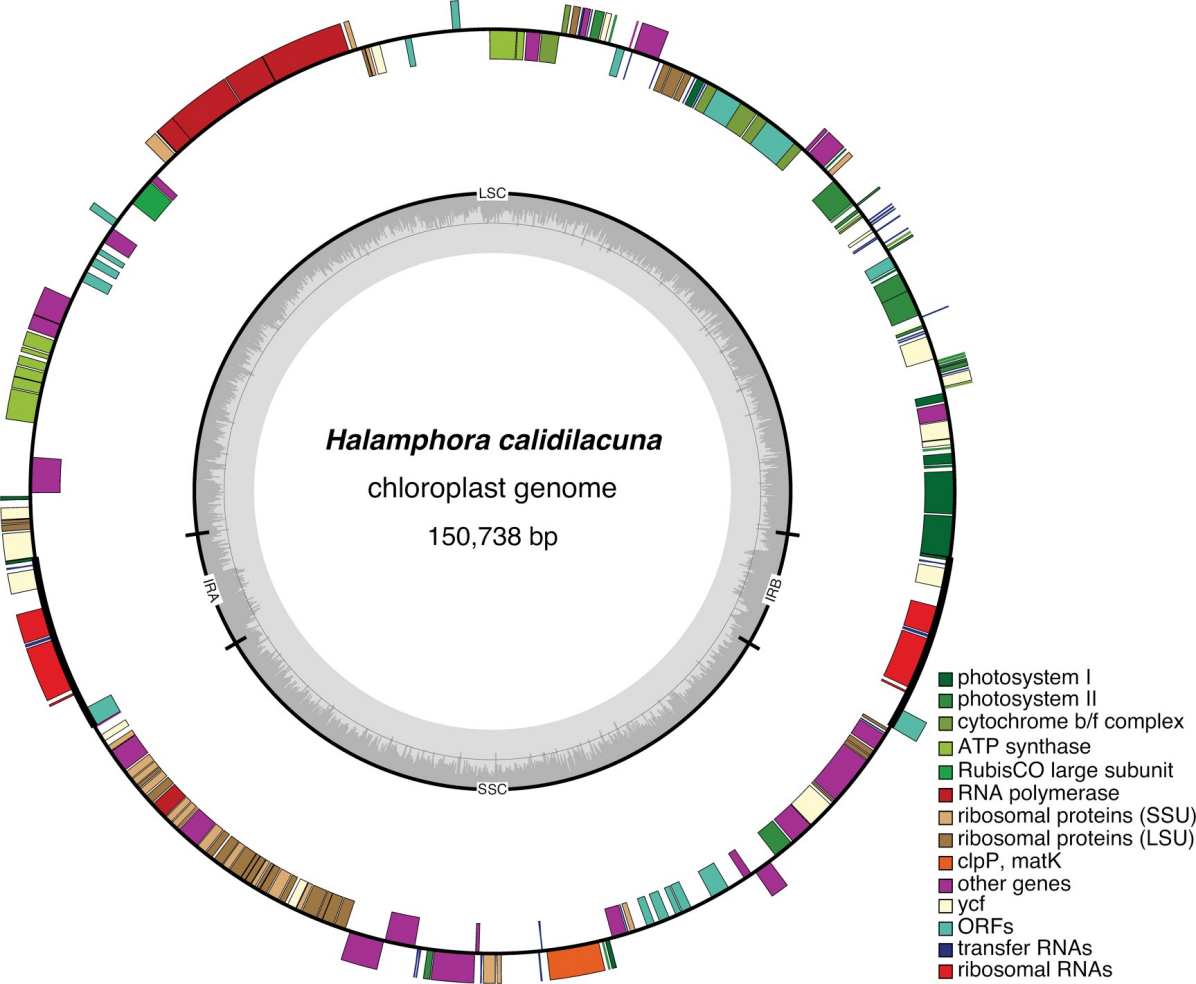
Plastid genome map of *Halamphora calidilacuna*. Genes on the outside are transcribed clockwise and those on the inside counterclockwise. The inner ring displays GC content in grey.

The three *Halamphora* genomes shared a set of 130 protein-coding genes, three rDNAs, 27 tRNAs, one tmRNA (transfer-messenger RNA) and ffs (signal recognition particle RNA) (Fig 1 and S1–S3 Figs) with a similar gene content to other pennate diatoms (S2 Table). With the exception of ORFs (see below), the chloroplast genomes of *H*. *americana*, *H*. *calidilacuna*, and *H*. *coffeaeformis* are nearly identical (99%) in gene content and differed only in the presence/absence of two genes– phosphoserine aminotransferase (*serC*) and cyclohexadienyl dehydrogenase (*tyrC*), both of which have been suggested to be of foreign or unknown origin, respectively [15].

Among the *Halamphora* genomes, *serC* was only present, and then only as a pseudogene, in *H*. *calidilacuna* and is presumed to be of plasmid origin. Plasmids have been found in only some pennate diatoms thus far [37,38]. *serC* has been found in five other diatom plastid genomes as a gene/pseudogene (S2 Table) and in plasmids of *Cylindrotheca* species [15,37,38]. An additional indication of the plasmid origin of this gene in *H*. *calidilacuna* is the presence of a partial (presumably non-functional) copy of ORF484 in the chloroplast genome, an ORF also found in the pCf2 plasmid of *C*. *fusiformis* [37,38].

**Fig 2:**
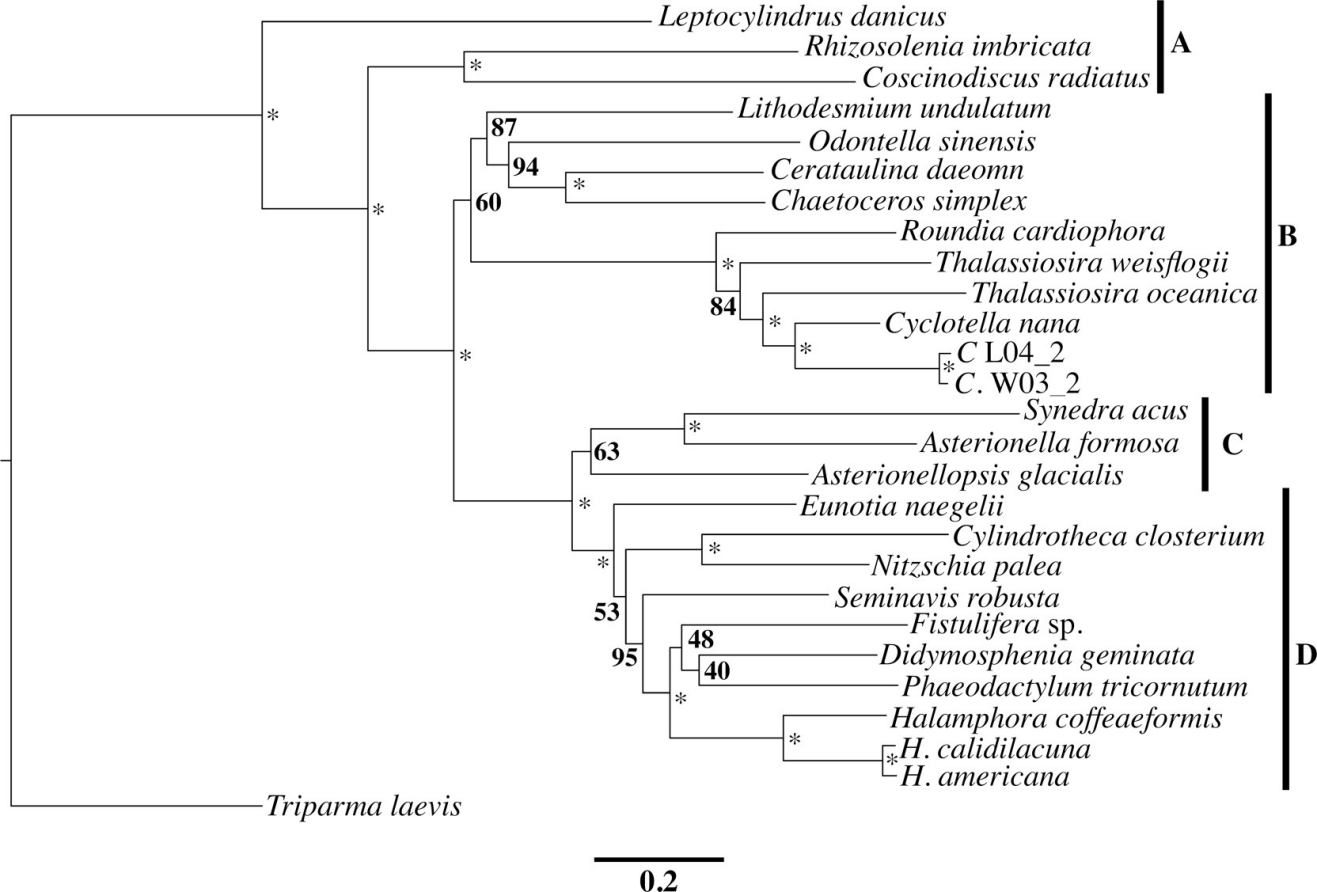
Maximum likelihood phylogram inferred from twenty chloroplast encoded markers (see Materials and methods). Node support is given as maximum likelihood bootstrap values (1000 bootstrap replicates). Asterisks indicate 100% support. Letters indicate morphological groups of diatoms as follows: (A) ‘radial’ centric; (B) ‘polar’ centric; (C) araphid; and (D) biraphid pennate diatoms.

The presence/absence of *tyrC* follows an interesting pattern, being present (and presumably functional) in *H*. *calidilacuna*, present and presumably non-functional pseudogene in *H*. *americana*, and absent in *H*. *coffeaeformis*. Although *tyrC* has also been found in other diatoms, green algae, and bacteria, its origin is less clear [15]. The presence as a gene/pseudogene in the two most closely related *Halamphora* taxa (Fig 2) and absence in the other taxon could indicate an acquisition via plasmid or from bacterial horizontal gene transfer (HGT) prior to the split of *H*. *calidilacuna* and *H*. *americana*, followed by a subsequent loss of function in *H*. *americana*. Alternatively, it is possible that all *Halamphora* species contain *tyrC*, but it is in transition to relocating to the nucleus.

Differences in genome size are due to difference in length of the IRs and intergenic regions (Table 2). Gene density (calculated as the number of protein coding genes / genome size) was inversely proportional to genome size, with the greatest gene density in the smallest genome and vice versa (Table 2).

The largest *Halamphora* genome (*H*. *calidilacuna*; Table 2) also contains more open reading frames (ORFs) than the other congeners. Three ORFs are shared between at least two *Halamphora* genomes, but no ORF is common to all three genomes (S2 Table). Only two ORFs (ORF385, ORF484) were shared between *Halamphora* species and other diatom genomes. ORF385, which was free standing in the genome, was found in *H*. *calidilacuna*, *Seminavis robusta*, and *Asterionellopsis glacialis*. A partial (presumably non-functional) copy of ORF484, also free standing, was found in *H*. *calidilacuna* and within a plasmid of *Cylindrotheca fusiformis* [37,38]. Similar to the retrotransposons found in diatom mitochondrial genomes [20], two group II introns containing regions with homology to reverse transcriptases/maturases (ORF26 & ORF27) were found in *H*. *calidilacuna* and *S*. *robusta*. In *H*. *calidilacuna*, ORF26 was present in an intron of *petD* and ORF27 was in an intron of *petB*. These two ORFs show some similarity (~70% similarity at > 86% coverage) to group II introns found in Ulvophytes [39] and red algae [33].

The nucleotide identity between sister taxa *H*. *calidilacuna* and *H*. *americana* is 98%, and the identity between either of these taxa and *H*. *coffeaeformis* is 88%. In addition to overall sequence similarity, the utility of the *rbcL* (~1400 bp) and *rbcL*-3P (748 bp) as barcode markers [40] to distinguish between these closely related taxa was also evaluated. Both *rbcL* barcode markers (both phylogenetic and shorter *rbc*L-3P lengths; [40]) show the same pattern as the overall similarity; 1% divergence was observed between *H*. *calidilacuna* and *H*. *americana* and 5–6% (*rbc*L and *rbc*L-3P, respectively) divergence was observed between *H*. *coffeaeformis* and either of these taxa. Therefore, either of these barcoding markers could be used to distinguish between these closely related *Halamphora* species.

Seven pairs of overlapping genes were found in one or more *Halamphora* species, with some overlapping pairs also found in the Thalassiosirales. Overlapping pairs found in both *Halamphora* spp. and Thalassiosirales include: *psbD*-*psbC* by 53 bp; *atpF*-*atpD* by 4 bp; and *rpl23*-*rpl4* by 11 bp in *Halamphora* spp. and 8–17 bp in the Thalassiosirales. Additional overlapping pairs include: *sufB*-*sufC* by 1 bp (*H*. *americana*, *H*. *calidilacuna*, and Thalassiosirales), *ycf45*-*psaB* by 4 bp (*H*. *coffeaeformis* and *H*. *calidilacuna*), within ORF25 by 15 bp (*H*. *americana*); and ORF385-*serC* by 2 bp (*H*. *calidilacuna*). Despite the widespread occurrence of overlapping genes, the origin, evolution and ramification of these overlaps remains unknown [41]. Some overlapping genes (e.g., *psbD*-*psbC*) are known to cause translational coupling, i.e, the translation of the *psbC* cistron depends on the translation of the *psbD* cistron [42]. Overlapping genes may also produce novel *de novo* proteins, a process common in viral genomes that can lead to changes in pathogenicity and possibly genome evolution [43]. Another type of alternative transcription (i.e., intron retention) is important to diatoms’ ability to adapt to changing nutrient conditions and not trivial in maintaining their physiology [44].

### Phylogeny results

The resulting phylogeny (Fig 2) revealed ‘polar’ centric (B), araphid (C), and biraphid pennate (D) diatoms to be monophyletic groups and ‘radial’ centric (A) diatoms to be a paraphyletic group. The monophyly of araphid diatoms is most likely due to the limited taxon sampling in our tree, as this group is often paraphyletic in analyses that include a larger number of taxa in this group [16,45,46]. The monophyly of ‘polar’ centric diatoms may be due to taxon sampling as well [16,45,46], but some studies have shown this group to be monophyletic (e.g., [47,48]) and this is an area of ongoing research. Within the biraphid pennate diatoms, the relationships between the genus *Halamphora* and the remaining taxa largely agrees with the multi-gene phylogenies of Ruck & Theriot [49] and Stepanek & Kociolek [13]. However, a single gene (18S rDNA) phylogeny presented by Zgrundo et al. [50], which includes taxa from the genus *Fistulifera* H. Lange-Bertalot, recovered this genus within a clade consistently more closely related to the *Halamphora* than to the rest of the biraphid pennate diatoms [13,49,50]. The 20-gene phylogeny presented here continues to strongly support (BS 100) the monophyly of the genus *Halamphora* as well as the close sister relationship between *H*. *americana* and *H*. *calidilacuna* that have been recovered in several broadly-sampled multigene phylogenies of the group [13,14].

### Synteny results

**Fig 3:**
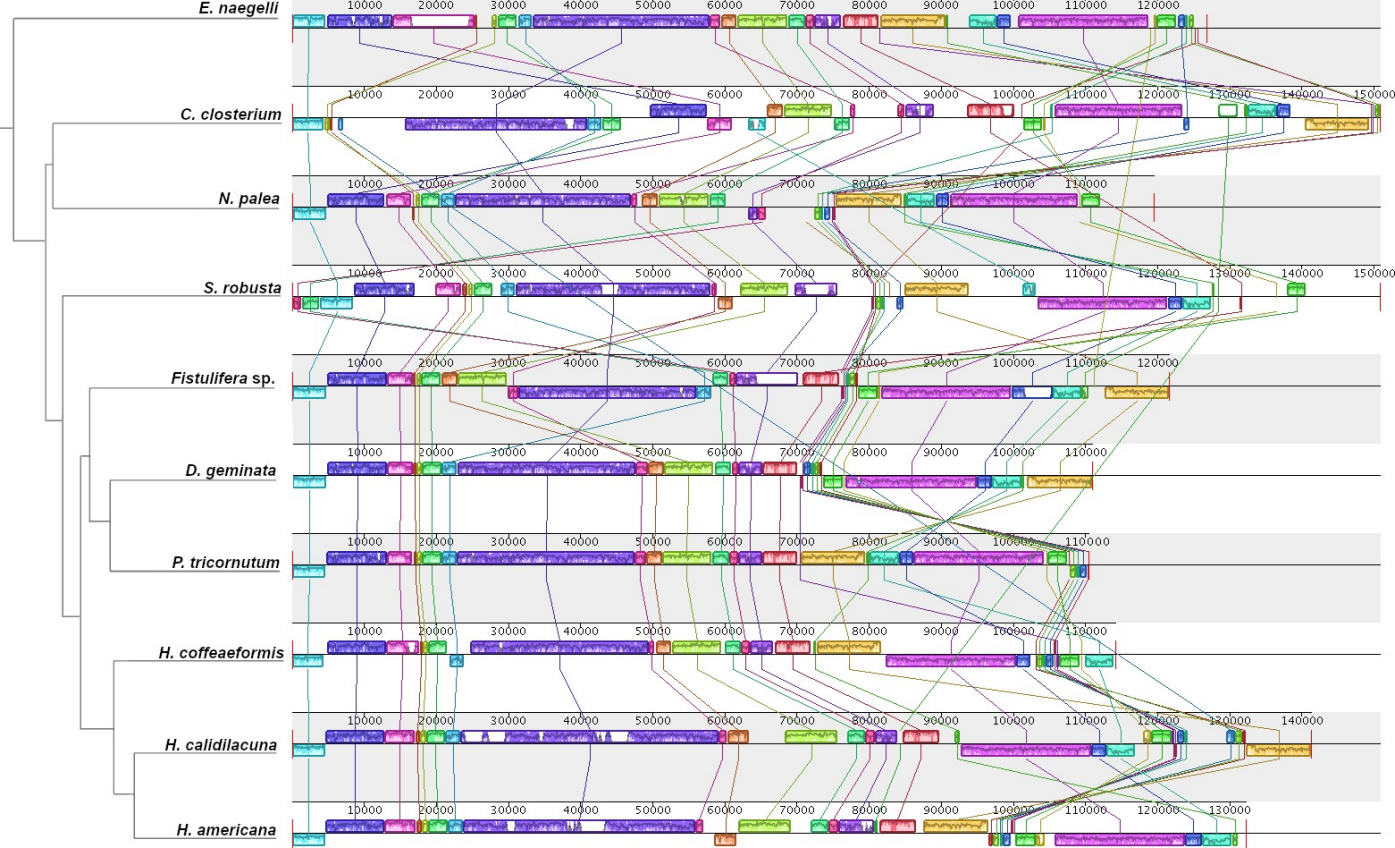
Gene order comparison of plastid genomes of eight biraphid pennate diatoms (three *Halamphora* spp. are from this study) with one copy of the inverted repeat removed prior to analysis. Alignment and resulting locally collinear blocks (LCBs) were generated using MAUVE. Relationships between taxa displayed as a cladogram to the left of the diagram are based on Fig 2.

The MAUVE alignment of biraphid pennate diatoms resulted in 26 locally collinear blocks (LCBs) of sequence among the ten diatoms examined (Fig 3). This gene order comparison revealed inversions, translocations, and inversion/translocation combinations resulting in extensive plastid genome rearrangement across this group (Fig 3). Distances between gene orders of LCBs calculated using GRIMM revealed the gene order of *Cylindrotheca closterium* to be the most unique among these diatoms (S3 Table). Although gene order was more conserved within the clade containing *Didymosphenia geminata*, *Phaeodactylum tricornutum*, *H*. *americana*, *H*. *calidilacuna*, and *H*. *coffeaeformis*, no chloroplast genomes in these analyses had identical gene order (Fig 3 and S3 Table). Within *Halamphora*, gene order was not conserved and distances between gene orders of LCBs were ≥ 3 (S3 Table).

**Fig 4:**
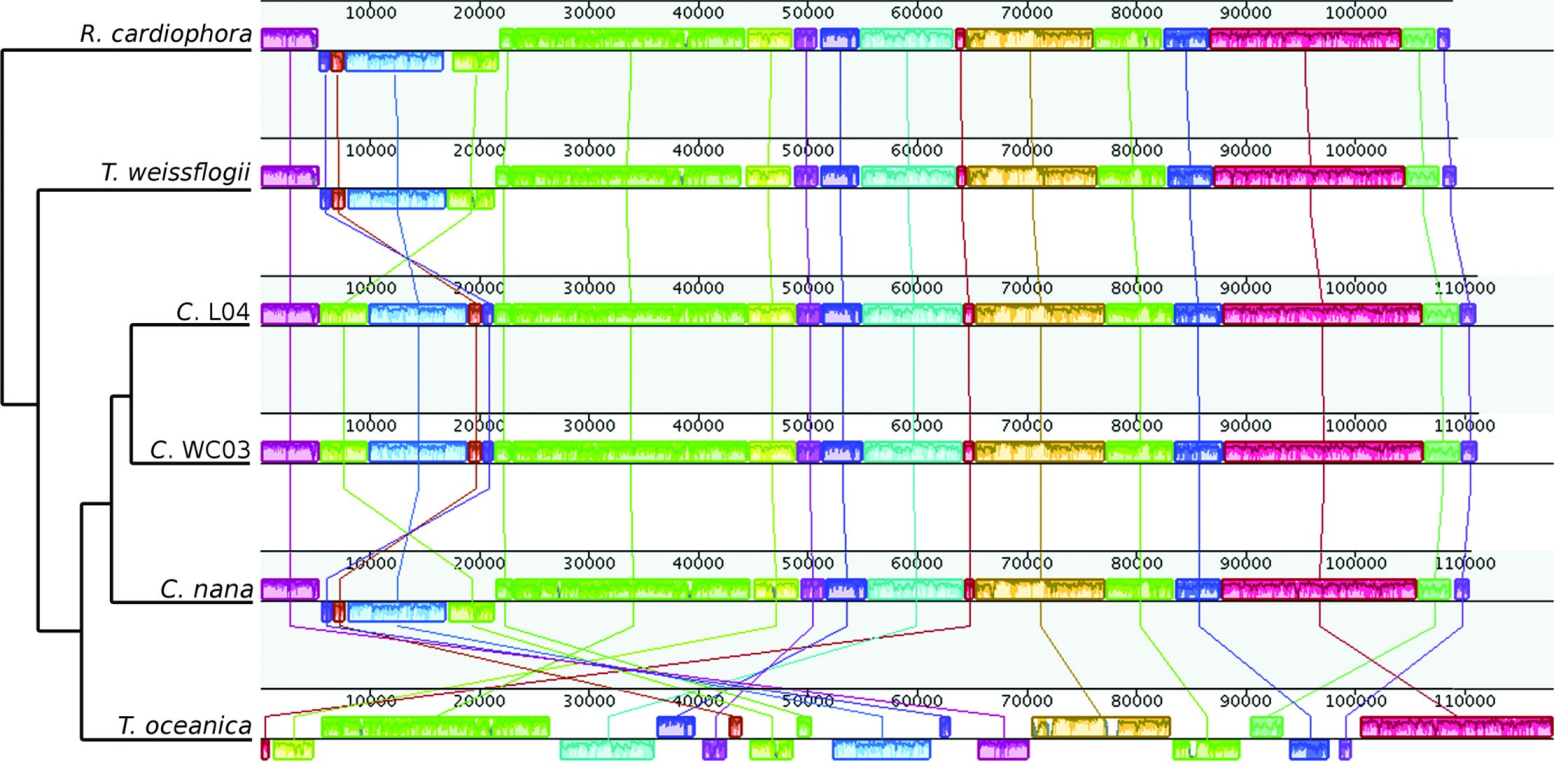
Gene order comparison of plastid genomes of six thalassiosiroid diatoms with one copy of the inverted repeat removed prior to analysis. Alignment and resulting locally collinear blocks (LCBs) were generated using MAUVE. Relationships between taxa displayed as a cladogram to the left of the diagram are based on Fig 2.

**Fig 5:**
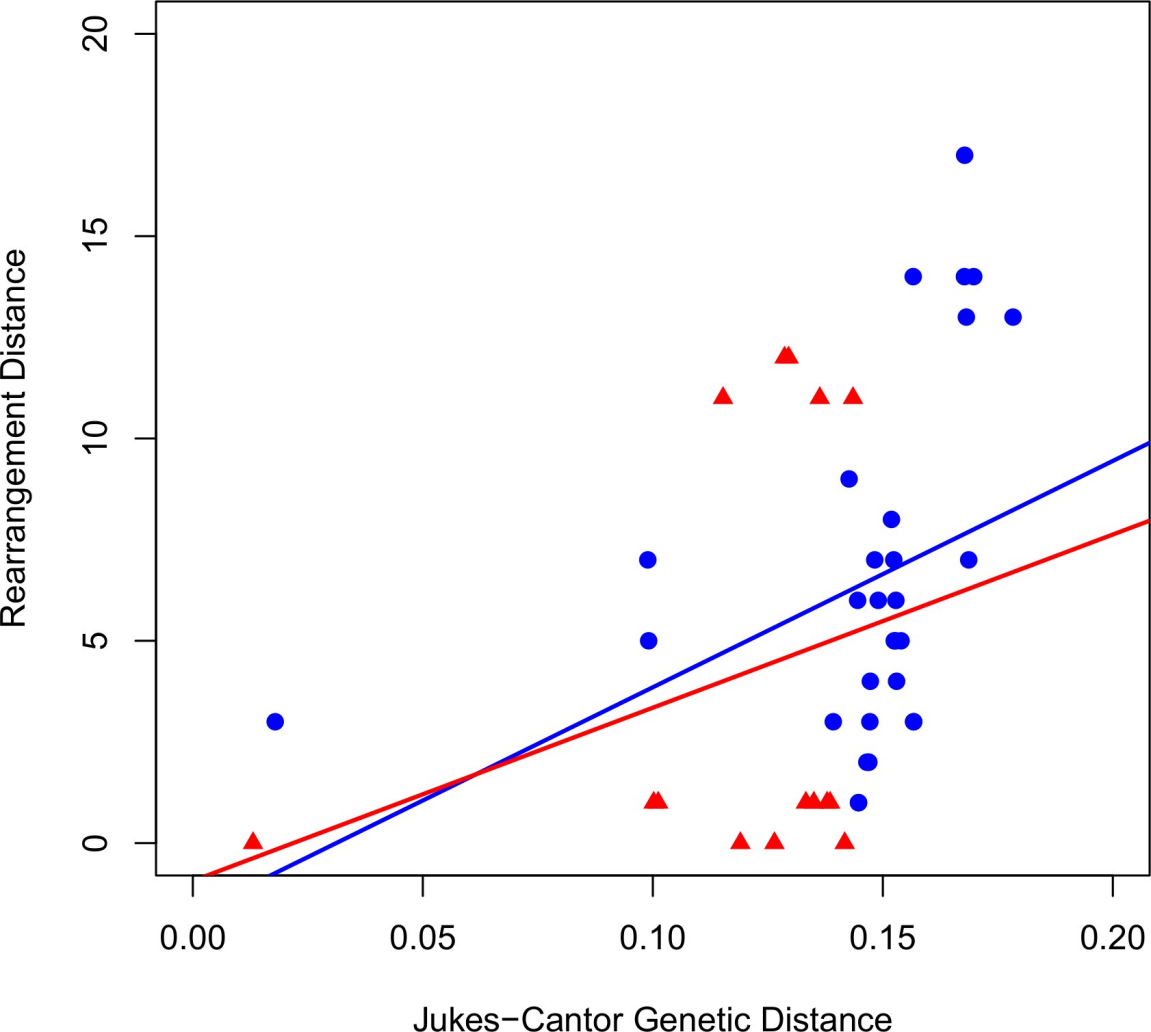
Plot describing the relationship between rearrangement distance and Jukes-Cantor genetic distance. Blue circles represent biraphid diatoms. Red triangles represent thalassiosiroid diatoms. Regression coefficient for the biraphid diatoms is significant (p = 0.04, R^2^ = 0.136), whereas for the thalassiosiroid diatoms it is not (p = 0.34, R^2^ = 0.069).

Gene order of the thalassiosiroid diatoms [15,16] was examined as a comparison to the rearrangements observed within the biraphid pennate diatoms including *Halamphora*. The MAUVE alignment of thalassiosiroid diatoms resulted in 18 locally collinear blocks (LCBs) of sequence among the six diatoms examined (Fig 4). With the exception of *T*. *oceanica*, gene order is more conserved in the thalassiosiroid diatoms (Fig 4) and the resulting rearrangement distances are smaller (S4 Table and Fig 5), a pattern consistent with the findings of Ruck et al. [15] and Sabir et al. [16]. In particular, there was only one inversion between *Cyclotella* species (Fig 4), in contrast to the inversions, translocations, and inversion/translocation combinations within *Halamphora* spp. (Fig 3). A comparison of Jukes-Cantor genetic distance to rearrangement distance generated in GRIMM for both biraphid and thalassiosiroid diatoms (Fig 5) shows a weak but positive relationship between these two measures of evolution.

The number of inversions and translocations inferred within a genus of diatoms is surprisingly high, particularly among species of *Halamphora*, where congeners may differ by up to 7 detectable rearrangements (S3 Table). This represents a striking contrast to chloroplast genomes in most land plants, where gene order is typically highly conserved within genera, and even across diverse families, orders, and in some cases classes [51]. For example, the monocot *Oryza sativa* differs from the dicot *Arabidopsis thaliana* by only one inversion and a translocation, despite those lineages diverging an estimated 160 million years ago [52]. The level of sequence divergence (12%), differences in gene content (2 genes differ in presence/absence), and degree of genome feature rearrangement (~7 rearrangements) found in *Halamphora* is high, and in order to recapitulate this level of divergence in land plants, one must compare as disparate groups as angiosperms (i.e., *A*. *thaliana*: accession NC_000932) and Equisetales (i.e., *Equisetum arvense*: accession NC_014699) [52]. Based on estimates in a recent publication [45], *Halamphora* evolved ~75–100 MYA, compared to over 400 MYA between angiosperms and Equisetales [53]. The first occurrence of amphoroid diatoms in the fossil record comes from the Oamaru Formation [54], estimated to be of Eocene age (33–55 MYA). These data suggest that chloroplast genomes of this genus are evolving approximately four (based on the estimates of the first occurrence of the group) to seven (based on occurrence in the fossil record) times faster than those of land plants in multiple ways, including sequence divergence, gene content, and gene order.

## Conclusions

Numerous studies have compared gene content and genome rearrangement among diatom chloroplast genomes (e.g., [15,16,17,32,55,56,57,58,59,60,61,62]), but this is the first to compare multiple genomes within a genus of biraphid pennate diatoms and several surprising patterns were identified. In regard to the phylogenetic position of *Halamphora* and the relationship between *Halamphora* species, our 20-gene phylogeny supported similar relationships to those revealed in other studies [13,14]. As with other diatoms, these chloroplast genomes are evolving relatively rapidly at the sequence level (12% divergence across the genus *Halamphora*). *Halamphora* plastid genomes also showed variation in gene content, with species incorporating two genes. Even more striking variation was observed in gene order, with multiple inversions, translocations, and inversion/translocation combinations found within this genus. *Cyclotella*, a thalassiosiroid genus, showed more conservation in gene order, with only one inversion. Overall, biraphid pennate diatoms appear to display more variation in gene order than the thalassiosiroid diatoms and significantly more variation than typical land plant chloroplasts (notable exceptions include the Geraniaceae family [63] and *Amborella* [64]). Although this pattern is conspicuous, only 0.04% of the estimated 100,000 diatom species’ chloroplast genomes have been examined and therefore, additional data and comparisons are necessary before generalizations should be made regarding overall diatom chloroplast genome evolution. Although these data are preliminary (comprising only a fraction of diatom diversity), they point to a comparable degree of variation within this one genus of diatoms to the divergence among distant divisions of vascular plants. This diversification within *Halmaphora* is accompanied by a substantially higher (4–7×) rate of evolution. These remarkable intrageneric and inter-kingdom comparisons require additional data to verify the results. However, if these data are supported by additional studies, they open the door to many questions about the rate and modes of molecular evolution of the chloroplast genome in this remarkable clade.

## Supporting information

S1 Table Diatom chloroplast genomes used as reference for annotating the *Halamphora* genomes. (DOCX)

S2 Table Gene content comparison of three *Halamphora* spp. genomes (bold) with other published diatom plastid genomes. X, present; P, pseudogenized copy of gene is present; -, absent.
(XLSX)

S3 Table Distances between gene orders of LCBs (generated in MAUVE) of biraphid pennate diatoms (taxa from this study are in bold) calculated using GRIMM. Smaller values indicate more similar gene order.
(DOCX)

S4 Table Distances between gene orders of LCBs (generated in MAUVE) of thalassiosiroid diatoms calculated using GRIMM. Smaller values indicate more similar gene order.
(DOCX)

S1 Fig Plastid genome map of *Halamphora calidilacuna*. Genes on the outside are transcribed clockwise and those on the inside counterclockwise. The inner ring displays GC content in grey.
(TIF)

S2 Fig Plastid genome map of *Halamphora americana*. Genes on the outside are transcribed clockwise and those on the inside counterclockwise. The inner ring displays GC content in grey.
(TIF)

S3 Fig Plastid genome map of *Halamphora coffeaeformis*. Genes on the outside are transcribed clockwise and those on the inside counterclockwise. The inner ring displays GC content in grey.
(TIF)

S1 File Fasta alignment. The fasta alignment of twenty molecular markers (*psaA*, *psbC*, *petD*, *petG*, *atpA*, *atpG*, *rbcL*, *rbcS*, *rpoA*, *rpoB*, *rps14*, *rpl33*, *rnl*, *rns*, *ycf89*, *sufB*, *sufC*, *dnaK*, *dnaB*, *clpC*) from 26 species used to generate the phylogeny presented in this study.
(FASTA)
