# Supplementary material for: Extensive chloroplast genome rearrangement amongst three closely related Halamphora spp. (Bacillariophyceae), and evidence for rapid evolution as compared to land plants
Source: PLoS One. 2019 Jul 3;14(7):e0217824. doi: 10.1371/journal.pone.0217824 (PMC6608930; doi:10.1371/journal.pone.0217824)
Supplement: S1 Table — (DOCX) [file pone.0217824.s001.docx]

**S1 Table. Diatom chloroplast genomes used as reference for annotating the *Halamphora* genomes.**

| Taxon | GenBank accessions | Publication |
| --- | --- | --- |
| *Asterionella formosa* | NC_024079 | Ruck et al. 2014 |
| *Asterionellopsis glacialis* | NC_024080 | Ruck et al. 2014 |
| *Cerataulina daemon* | KJ958484 | Sabir et al. 2014 |
| *Chaetoceras simplex* | NC_025310 | Sabir et al. 2014 |
| *Coscinodiscus radiatus* | NC_024081 | Ruck et al. 2014 |
| *Cyclotella nana* | EF067921 | Oudot-Le Secq et al. 2007 |
| *Cyclotella* sp. L04_2 | KJ958480 | Sabir et al. 2014 |
| *Cyclotella* sp. WC03_2 | KJ958481 | Sabir et al. 2014 |
| *Cylindrotheca closterium* | NC_024082 | Ruck et al. 2014 |
| *Didymosphenia geminata* | NC_024083 | Ruck et al. 2014 |
| *Durinskia baltica* | NC_014287 | Imanian et al. 2010 |
| *Eunotia naegelii* | NC_024928 | Ruck et al. 2014 |
| *Fistulifera solaris* | AP011960 | Tanaka et al. 2011 |
| *Kryptoperidinium foliaceum* | GU591328 | Imanian et al. 2010 |
| *Leptocylindrus danicus* | NC_024084 | Ruck et al. 2014 |
| *Lithedesmium undulatum* | NC_024085 | Ruck et al. 2014 |
| *Nitzschia palea* | MH113811 | Crowell et al. 2018 |
| *Odontella sinensis* | NC_001713 | Kowallik et al. 1995 |
| *Phaeodactylum tricornutum* | EF067920 | Oudot-Le Secq et al. 2007 |
| *Rhizosolenia imbricata* | NC_025311 | Sabir et al. 2014 |
| *Roundia cardiopohra* | KJ958483 | Sabir et al. 2014 |
| *Seminavis robusta* | MH356727 | Brembu et al. 2014 |
| *Synedra acus* subsp. *radians* | NC_016731 | Galachyants et al. 2015 |
| *Thalassiosira oceanica* | GU323224 | Lommer et al. 2010 |
| *Thalassiosira weissflogii* | NC_025314 | Sabir et al. 2014 |
