# Supplementary material for: Extensive chloroplast genome rearrangement amongst three closely related Halamphora spp. (Bacillariophyceae), and evidence for rapid evolution as compared to land plants
Source: PLoS One. 2019 Jul 3;14(7):e0217824. doi: 10.1371/journal.pone.0217824 (PMC6608930; doi:10.1371/journal.pone.0217824)
Supplement: S3 Table — Smaller values indicate more similar gene order. (DOCX) [file pone.0217824.s003.docx]

**S3 Table. Distances between gene orders of LCBs (generated in MAUVE) of biraphid pennate diatoms (taxa from this study are in bold) calculated using GRIMM.** Smaller values indicate more similar gene order.

|  | *Eunotia naegelii* | *Cylindrotheca*  *closterium* | *Nitzschia palea* | *Seminavis robusta* | *Fistulifera* sp. | *Didymosphenia*  *geminata* | *Phaeodactylum*  *tricornutum* | ***Halamphora***  ***coffeaeformis*** | ***H. calidilacuna*** | ***H. americana*** |
| --- | --- | --- | --- | --- | --- | --- | --- | --- | --- | --- |
| *Eunotia naegelii* | - | 14 | 8 | 9 | 5 | 3 | 2 | **9** | **6** | **7** |
| *Cylindrotheca closterium* | 14 | - | 14 | 19 | 13 | 13 | 14 | **17** | **14** | **14** |
| *Nitzschia palea* | 8 | 14 | - | 9 | 8 | 6 | 7 | **10** | **7** | **8** |
| *Seminavis robusta* | 9 | 19 | 9 | - | 10 | 8 | 9 | **10** | **10** | **9** |
| *Fistulifera* sp. | 5 | 13 | 8 | 10 | - | 2 | 3 | **8** | **5** | **6** |
| *Didymosphenia geminata* | 3 | 13 | 6 | 8 | 2 | - | 1 | **6** | **3** | **4** |
| *Phaeodactylum tricornutum* | 2 | 14 | 7 | 9 | 3 | 1 | - | **7** | **4** | **5** |
| ***Halamphora coffeaeformis*** | **9** | **17** | **10** | **10** | **8** | **6** | **7** | **-** | **5** | **7** |
| ***H. calidilacuna*** | **6** | **14** | **7** | **10** | **5** | **3** | **4** | **5** | **-** | **3** |
| ***H. americana*** | **7** | **14** | **8** | **9** | **6** | **4** | **5** | **7** | **3** | **-** |
