# Supplementary material for: Extensive chloroplast genome rearrangement amongst three closely related Halamphora spp. (Bacillariophyceae), and evidence for rapid evolution as compared to land plants
Source: PLoS One. 2019 Jul 3;14(7):e0217824. doi: 10.1371/journal.pone.0217824 (PMC6608930; doi:10.1371/journal.pone.0217824)
Supplement: S4 Table — Smaller values indicate more similar gene order. (DOCX) [file pone.0217824.s004.docx]

**S4 Table. Distances between gene orders of LCBs (generated in MAUVE) of thalassiosiroid diatoms calculated using GRIMM.** Smaller values indicate more similar gene order.

|  | *Roundia*  *cardiophora* | *Thalassiosira*  *weissflogii* | *Cyclotella* sp.  L04_2 | *Cyclotella* sp.  W03_2 | *C. nana* | *T. oceanica* |
| --- | --- | --- | --- | --- | --- | --- |
| *Roundia cardiophora* | - | 0 | 1 | 1 | 0 | 11 |
| *Thalassiosira weissflogii* | 0 | - | 1 | 1 | 0 | 11 |
| *Cyclotella* sp. L04_2 | 1 | 1 | - | 0 | 1 | 12 |
| *Cyclotella* sp. W03_2 | 1 | 1 | 0 | - | 1 | 12 |
| *C. nana* | 0 | 0 | 1 | 1 | - | 11 |
| *T. oceanica* | 11 | 11 | 12 | 12 | 11 | - |
