# Supplementary figures and images for: Extensive chloroplast genome rearrangement amongst three closely related Halamphora spp. (Bacillariophyceae), and evidence for rapid evolution as compared to land plants
Source: PLoS One. 2019 Jul 3;14(7):e0217824. doi: 10.1371/journal.pone.0217824 (PMC6608930; doi:10.1371/journal.pone.0217824)

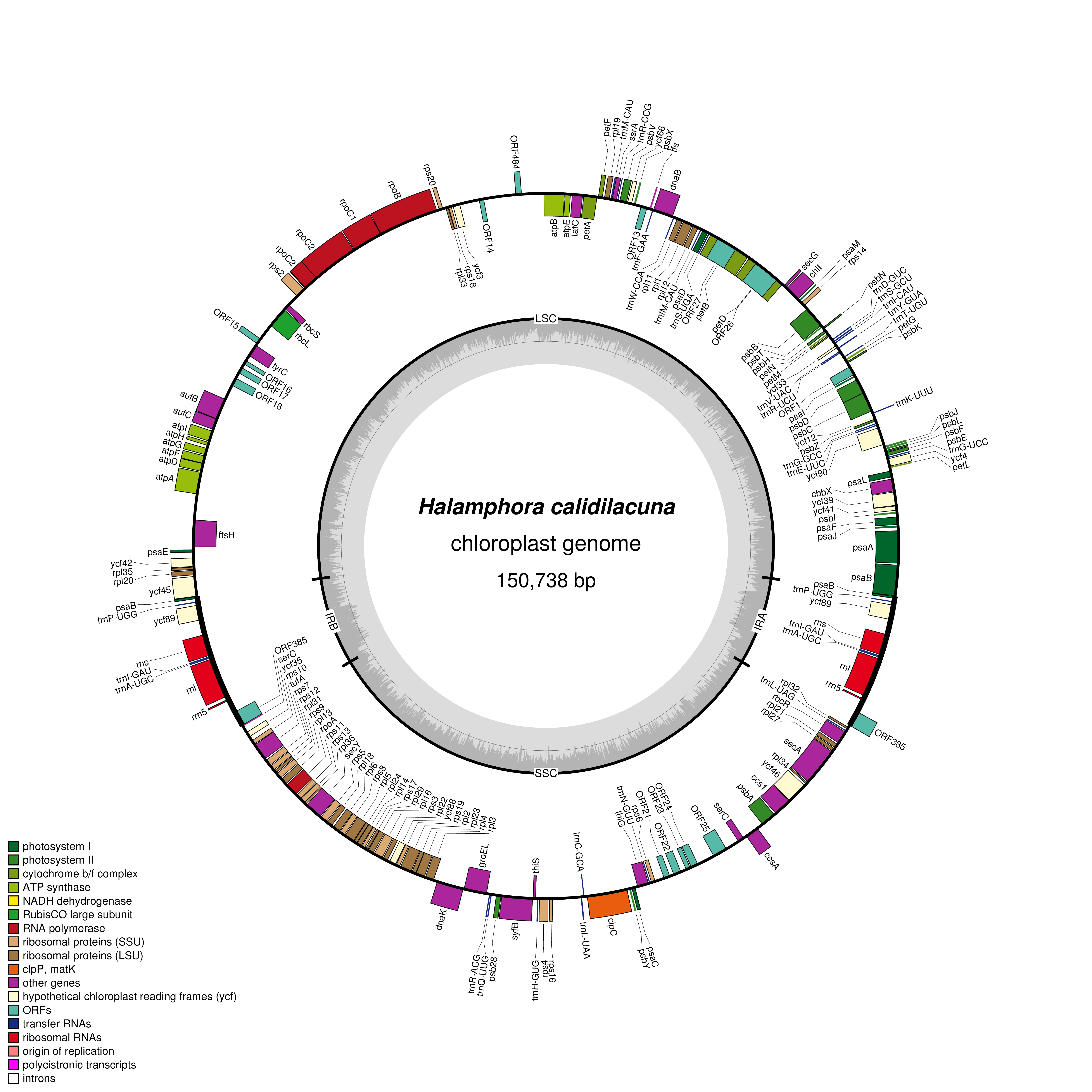

Supplement: S1 Fig — Genes on the outside are transcribed clockwise and those on the inside counterclockwise. The inner ring displays GC content in grey. (TIF) [file pone.0217824.s005.tif]
